# Clinical and histological evaluation of increase in the residual ridge width using mineralized corticocancellous block allografts: A pilot study

**DOI:** 10.15171/joddd.2017.040

**Published:** 2017-12-13

**Authors:** Reza Shahmohammadi, Amir Moeintaghavi, Mehrdad Radvar, Habibollah Ghanbari, Nasrollah Saghravanian, Shabnam Aghayan, Sara Sarvari

**Affiliations:** ^1^Department of Endodontics, Faculty of Dentistry, Mashhad University of Medical Sciences, Mashhad, Iran; ^2^Dental Material Research Center, Mashhad University of Medical Sciences, Mashhad, Iran; ^3^Department of Periodontics, Faculty of Dentistry, Mashhad University of Medical Sciences, Mashhad, Iran; ^4^Oral and Maxillofacial Research Center, Mashhad University of Medical Sciences, Mashhad, Iran; ^5^Department of Periodontics, Dental Branch, Islamic Azad University (Tehran), Tehran, Iran; ^6^Mashhad University of Medical Sciences, Mashhad, Iran

**Keywords:** Allografts, alveolar ridge augmentation, dental implants

## Abstract

***Background.*** Lateral ridge augmentation is conventionally accomplished by means of autogenous bone grafts. However, due to its complications, the application of autogenous bone graft substitutes, e.g. mineralized corticocancellous allograft, is ecommended.

***Methods.*** In the present study, twelve patients were included, with insufficient alveolar ridge widths in the designated sites for dental implant placement. During the primary surgery, mineralized corticocancellous block allografts were fixed in deficient sites with titanium screws and resorbable collagen membranes were used to cover the blocks. After a period of six months, a flap was raised and variations in ridge width values was measured. Finally, a micro-biopsy was obtained from the sites for histologic investigation prior to preparing them for subsequent implant placement.

***Results.*** All the applied blocks were incorporated into the underlying bone except for one. A statistically significant difference was seen between the average ridge widths before placing the allografts compared with that of implant placement stage (2.62±1.02 mm vs. 7.75±1.63 mm, respectively). Vital bone tissue was detected in all the histological specimens obtained from the interface of blocks and the underlying bone.

***Conclusion.*** The results suggest that mineralized corticocancellous block allografts might be used as scaffolds for bone growth and ridge width augmentation.

## Introduction

Dental implants offer an alternative for missing teeth to restore natural ridge contours for esthetics and support of a dental prosthesis.^1^ The success of such treatment relies heavily upon the presence of sufficient bone volume in the treatment site and full bone coverage, which ensure reliable osteointegration.^2-10^

In case of deficient alveolar ridge, alveolar bone volume should be increased to an adequate level prior to implant placement so that longer and wider implants could be placed in a stable position.^6^ Various surgical techniques have been proposed so far to augment the deficient ridge. Guided Bone Regeneration (GBR) is one of these techniques, which involves the application of a membrane in order to retract the soft tissue cells and promote formation of new bone.^11^ However, membranes often fail to maintain the space for osteosynthesis and therefore, several graft materials have been proposed to support them.^11^ Autogenous bone graft currently serves as a predictable method of bone augmentation.^12-16^ Autogenous grafts not only provide a bed for osteoconduction, but also possess osteoinductive characteristics through their vital cells capable of bone formation.^11^ Nevertheless, harvesting autogenous bone ‒ both intraorally and extraorally ‒ is considered to be unpleasant for the patients and may be associated with various complications such as paresthesia, facial deformity and drooping of the chin.^17-22^ In addition, obtaining large amounts of autogenous bone to compensate for relatively large alveolar defects is practically impossible, especially when there is inadequate bone volume in intraoral donor sites.^23^ Therefore, in order to eliminate the need for a second surgical donor site, the application of block allografts is recommended.^24,25^ It should be noted that no adverse systemic incidents have occurred so far in terms of cross-infection (e.g. hepatitis or HIV), with the advent of the new guidelines, which has increased the popularity of allografts.^26,27^

Recently, several laboratories in Iran have commenced the production of mineralized corticocancellous block allografts. In view of the scarcity of published data on the efficacy of this specific type of allograft, which is locally produced with a considerably lower price than its foreign counterparts, this study was conducted to evaluate the clinical and histological outcomes of a mineralized corticocancellous block allograft in combination with a resorbable membrane in the treatment of lateral ridge defects.

## Methods

**Figure 1:**
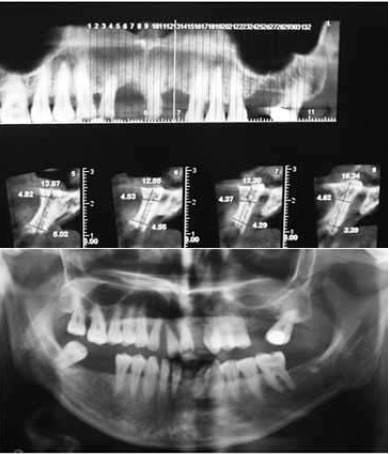
Preoperative radiographs.

**Figure 2:**
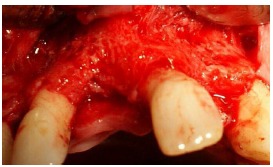
Cortical bone drilled in several spots.

The study was approved by the Ethics Committee of Mashhad University of Medical Sciences (MUMS) and was registered in the Iranian Registry of Clinical Trials (IRCT) database (IRCT num.: IRCT138812081601N2). Twelve female patients, 30‒60 years of age, with insufficient ridge width, were included in this clinical trial and informed consent was obtained from all the patients. In all the cases, bone grafting was needed in the anterior and premolar region to restore natural ridge contour to support an implant. All the patients had ridge widths measuring ≤3 mm, which would be amenable only to block grafting procedures rather than particulate grafts. Figure 1 shows preoperative radiographs of one of the patients. Patients' systemic health was good and there were no contraindications for surgical placement of implants. The exclusion criteria were limited to systemic conditions such as bisphosphonate use, which contraindicate surgical implant treatment. Two days prior to the surgery, a ten-day regimen of co-amoxiclav (625 mg) was administered. The patients were instructed to use chlorhexidine mouthwash 0.2% just before the operation. Under local anesthesia, a mucoperiosteal flap was elevated to clearly expose the ridge defect. Using a gauge, the width of the residual ridge was measured in its thinnest portion and then, several perforations were made into the cortical bone by a round bur (Figure 2).

**Figure 3:**
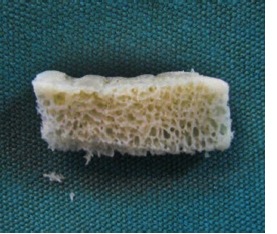
Mineralized corticocancellous block allograft.

**Figure 4:**
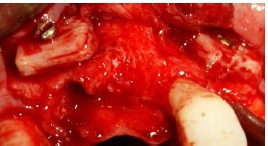
Block allograft secured in position by titanium screw.

Subsequently, the mineralized corticocancellous block allografts (Hamanandsaz, LIDCO, Kish, Iran) were hydrated with saline, trimmed to fit the defect size and fixed in place with titanium screws (Figures 3 and 4).

**Figure 5:**
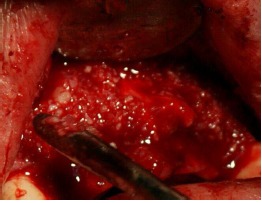
Residual gaps filled with particulate allograft material.

Residual gaps between the blocks and the underlying bone were filled with particulate allograft material, i.e. demineralized freeze-dried bone allograft (DFDBA) produced by the above-mentioned company (Figure 5).

**Figure 6:**
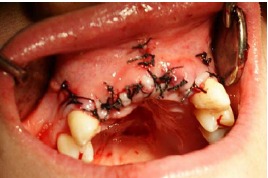
Mucosal flap closed using 3-0 non-resorbable suture.

**Figure 7:**
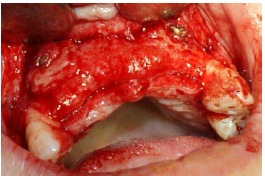
Surgical site reopened six months after augmentation for implant placement.

**Figure 8:**
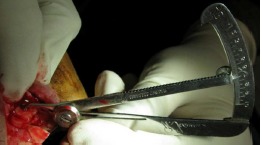
Ridge width measurement following six-month reentry.

The graft was covered with resorbable collagen membrane (Geistlich Bioguide pharma AG, Switzerland). The mucosal flap was closed using 3-0 non-resorbable (Supa, Tehran, Iran) sutures (Figure 6). To reduce postoperative inflammation, two doses of dexamethasone (8 mg and 4 mg) were administered immediately after and 12 hours after surgery, respectively. The patients also received acetaminophen to provide analgesic benefits. The sutures were removed 10 days later. Six months after the surgery, the flap was reopened (Figure 7) and the ridge width was measured in the same place using a gauge (Figure 8). This was carried out close to where the first measurement was made.

**Figure 9:**
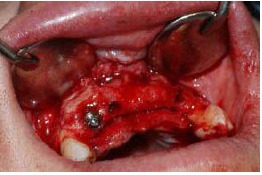
Biopsy sample taken from the implant drill site.

During implant placement, using a 3-mm trephine bur, a biopsy sample was taken from the interface of bone and allograft for histological assessment (Figure 9). All the samples were kept in 10% formalin solution and sent to a pathologist. In the laboratory, the samples were placed in decalcifying solution and then embedded in paraffin blocks. Following preparation of 4‒5-µm cross-sections, the blocks were stained with hematoxylin and eosin. Finally, the samples were histologically assessed to see whether a distinction could be made between the native bone and the newly-formed bone. Given that such a distinction is present, the samples would also be histologically investigated in terms of the amount of newly-formed bone, bone marrow spaces, the amount of corticocancellous block remaining and the degree of inflammation by a pathologist.

Using the Kolmogorov–Smirnov test, it was found that the conditions to employ a parametric test were met. Thus, paired t-test was chosen to compare the postsurgical ridge widths with their baseline values.

## Results

Of a total of 12 patients assessed in our study, in one case the corticocancellous allograft was detached from the bone after three weeks due to the loss of keratinized gingiva and had to be removed.

Prior to placement of the allografts, the mean ridge width was 2.62±1.02 mm, which increased to 7.75±1.02 mm six months afterwards just before implant placement. A statistically significant increase (P<0.001) was found between baseline and the six-month reentry using the paired t-test analysis (Table 1).

**Table 1:** Baseline ridge width in comparison with that of six-month re-entry

	**Baseline**		**Reentry**		**Changes**		**P**
**Ridge width (mm)**	Mean	SD	Mean	SD	Mean	SD	
	2.672	1.026	7.754	1.633	5.081	0.947	0.000

**Figure 10:**
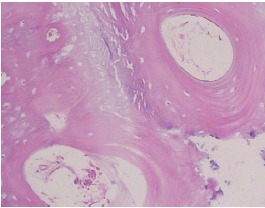
Illustration of a prepared histological cross-section.

Our histological analysis failed to reveal any clear distinction between the newly-formed bone and the native bone and no sign of allograft was detected in any of the prepared specimens. On the basis of this observation, no further histological investigation was performed regarding the amount of newly-formed bone, bone marrow spaces, the amount of corticocancellous block remaining and the degree of inflammation. Vital lamellar and trabecular bone tissue containing osteocytes and lacunar spaces were detected in the biopsy specimens (Figure 10).

## Discussion

The aim of this study was to evaluate the clinical results of lateral ridge augmentation technique in 12 female patients with insufficient ridge width for implant placement using corticocancellous block allografts and resorbable barrier membranes. The fact that all our patients were female was a coincidence but might be attributed to their higher interest in esthetic treatments. One of the 12 sites was excluded due to early exposure. It should be noted that the early exposure occurred in a region that two blocks were placed side-by-side with a gap of one tooth. It might be attributed to extreme tension of the flap in the mentioned block or excessive trauma caused by fixing the block in that area. Others^6^ have also reported identical failure of graft in their studies. In a study by Barone’s,^28^ two of the 24 allograft blocks were excluded due to early exposure. Likewise, in a study by Nissan's,^6^ soft tissue breakdown and graft exposure occurred in 28% of the cases.

In the present study, six months after graft placement, average ridge width gain was 5.081±0.947 mm, which was statistically significant. Lyford et al^11^ treated five deficient maxillary sites in 3 patients using freeze-dried cancellous block allograft and a resorbable membrane. They detected an increase of 2‒4 mm following the six-month re-entry and recommended cancellous block allograft as a viable alternative in order to avoid the complications of autogenous grafts.

No sign of infection or paresthesia was present in the augmentation sites and all the patients showed normal postoperative healing. The application of allograft avoided potential adverse complications associated with the donor site. Autogenous grafts may lead to sensory disturbance of soft tissues and formation of apical lesions in the adjacent teeth.^29^ Additionally, in some cases complete healing may not take place in the donor site.^29^ In view of these benefits, block allografts might be considered as an alternative to autogenous grafts.

The block allografts were completely attached to the underlying bone after 6 months. However, gaps were present in some regions, which was probably the result of rigidity and irregular shape of the allografts, making it difficult to adjust them suitably to the area they were going to be placed upon. Although prior to implant placement the gaps were filled with DFDBA, still some minor unfilled areas were probably present. Leonetti et al^20^ also observed full attachment of allografts to the bone along with some minor resorption.

In all the prepared histological cross-sections, allografts fused into the bone and no trace of the allograft was detected. Others^31^ have also shown successful incorporation of the allograft to the bone without any adverse complications, e.g. necrosis or inflammation. Vital lamellar and trabecular bone tissue with osteocytes and lacunar spaces were detected in the samples, which substantiates the growth of the underlying bone into the allograft. It should be mentioned that there was a limitation in the size of biopsy samples due to subsequent implant placement, which prevented a thorough histological investigation of the allografts in their entirety. Furthermore, the native bone was considerably thin and in some cases measured less than 2 mm, a fact which might have made us prepare implant sites entirely in the newly-formed bone, making the harvested specimens completely made of the new bone and containing no native bone holes so as not to compromise ridge integrity for implant placement. Consequently, the prepared holes might have not been large enough to reveal the interface of the two types of bone properly. Furthermore, harvesting a bone core biopsy through the buccal plate would have created a fenestration defect in the buccal aspect of implants, unnecessarily jeopardizing osseointegration as well as the esthetic outcome of our treatment, which is ethically unacceptable. In their histomorphometric analysis, Feuille et al^30^ reported that there was 47.6% new bone, while the residual allograft comprised 52.4%.

To the best of our knowledge, this pilot study is one of the first in Iran. Due to the cost of the treatment as well as patient reluctance, there was a limit in the number of subjects included in this survey. It is highly recommended that further studies be carried out in this field with more subjects and longer follow-up periods to evaluate the effectiveness of this method for patients with alveolar ridge defects in need of dental implant placement.

## Conclusion

Mineralized corticocancellous block allograft could be utilized as a scaffold to induce new bone formation and increase the alveolar ridge width. However, further research with longer follow-up evaluations is required to determine all the effective factors.

## Acknowledgments

We are most grateful to the administration of the School of Dentistry for the permission to report this case. Our sincere gratitude is extended to the patient for availing all the clinical information. We greatly appreciate the keenness of Miss Josephine W. Dwaluma in preparing this manuscript.

## Authors’ contributions

JM and KS performed the clinical and radiographic examinations, and drafted the manuscript.MC carried out a critical revision of the manuscript. All the authors contributed to final critical revision of the manuscript, and have read and approved the final manuscript.

## Funding

The authors report no funding for this article.

## Competing interests

The authors declare no competing interests with regards to the authorship and/or publication of this article.

## Ethics approval

The study was approved by the Ethics Committee of Mashhad University of Medical Sciences (MUMS) and was registered in the Iranian Registry of Clinical Trials (IRCT) database (IRCT No.: IRCT138812081601N2). The authors declare that the individual whose data is reported in this article has given consent to the authors for the publication of this report.
